# Detecting global form: separate processes required for Glass and radial frequency patterns

**DOI:** 10.3389/fncom.2013.00053

**Published:** 2013-05-08

**Authors:** David R. Badcock, Renita A. Almeida, J. Edwin Dickinson

**Affiliations:** School of Psychology (M304), The University of Western AustraliaCrawley, WA, Australia

**Keywords:** texture perception, form perception, human vision, global form, psychophysics, computational model

## Abstract

Global processing of form information has been studied extensively using both Glass and radial frequency (RF) patterns. Models, with common early stages, have been proposed for the detection of properties of both pattern types but human performance has not been examined to determine whether the two pattern types interact in the manner this would suggest. The experiments here investigated whether low RF patterns and concentric Glass patterns, which are thought to tap the same level of processing in form-vision, are detected by a common mechanism. Six observers participated in two series of masking experiments. First: sensitivity to the presence of either coherent structure, or contour deformation, was assessed. The computational model predicted that detection of one pattern would be masked by the other. Second: a further experiment examined position coding. The model predicted that localizing the center of form in a Glass pattern would be affected by the presence of an RF pattern: sensitivity to a change of location should be reduced and the apparent location should be drawn toward the center of the masking pattern. However, the results observed in all experiments were inconsistent with the interaction predicted by the models, suggesting that separate neural mechanisms for global processing of signal are required to process these two patterns, and also indicating that the models need to be altered to preclude the interactions that were predicted but not obtained.

## Introduction

**Figure 1:**
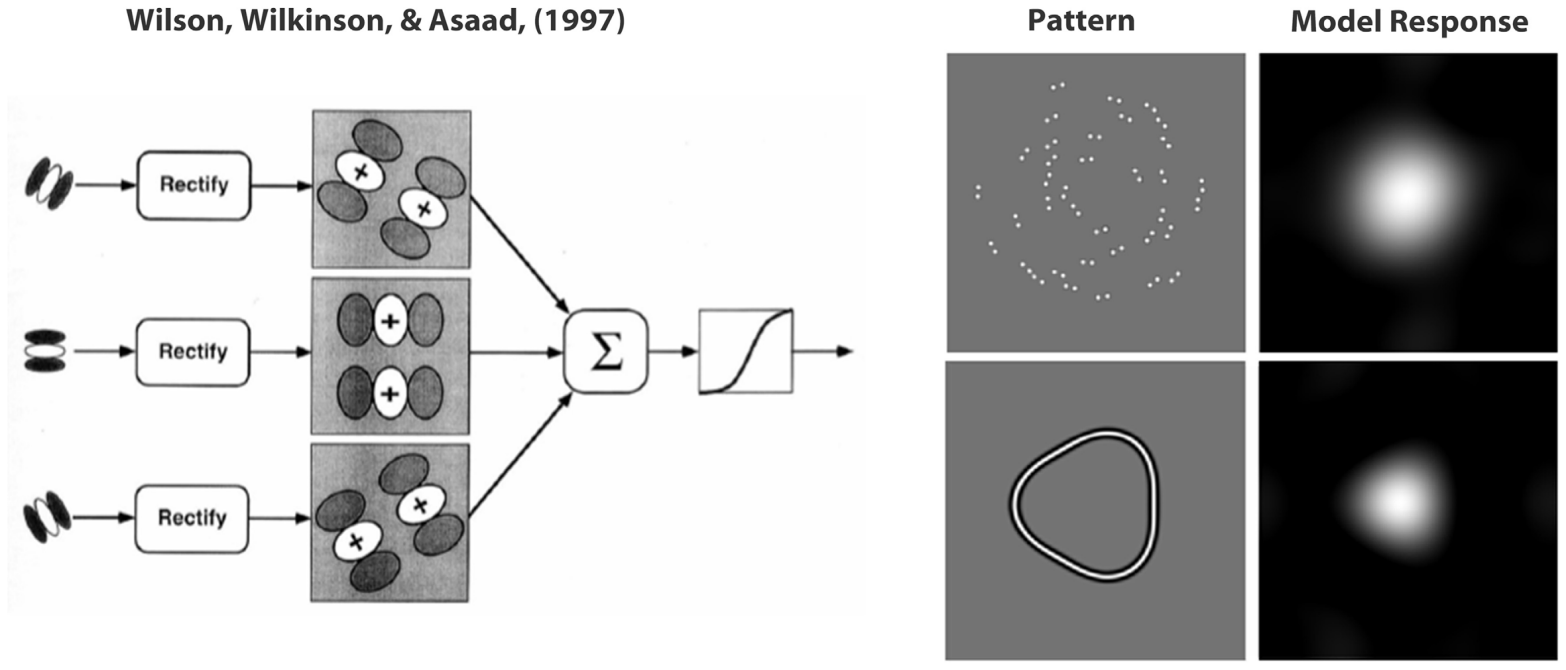
**The left hand side is a schematic copied from Wilson et al. (1997) representing their model.** The output of a family of oriented filters at the initial filtering stage is rectified and then re-filtered at a lower spatial frequency by a pair of filters orthogonally oriented relative to their specific initial filter but centered on the same spatial location. This output is then summed across all orientations and passed through a transducer function. The filtering is modeled as a convolution and therefore the response indicates the output obtained when the filters are centered on particular image locations. The right hand side shows an example Glass pattern (concentric 100% coherent) and an RF3 (lower). The model response to the patterns sits on their right hand side and the brightness indicates the strength of the response when the filter set is centered on the particular pixel. Brighter regions indicate stronger responses.

The visual system is adept at detecting pattern information consistent with global form; forms which frequently correspond to objects within a scene. Such global structures are commonly signaled either by coherence in the local texture within the object or by a clear outlining contour. Human detection of global structure has been studied using a number of different types of pattern. Some have used Glass patterns which are created by randomly scattering dot pairs throughout the image and then assigning a proportion of those pairs orientations that are consistent with a globally coherent structure (Glass, 1969; Dakin, 1997; Wilson and Wilkinson, 1998; Wilson et al., 2004; Badcock et al., 2005; Clifford and Weston, 2005; Mandelli and Kiper, 2005; Badcock and Clifford, 2006; Burr and Ross, 2006; Smith et al., 2007) while others have used radial frequency (RF) patterns in which a closed contour is systematically varied in shape by sinusoidally modulating the radius as a function of polar angle (Loffler et al., 2003; Bell et al., 2007; Loffler, 2008). Both of these patterns types have been used to show global accumulation of local signals and the detection and recognition processes for both types of pattern have been modeled (Wilson et al., 1997; Wilson and Wilkinson, 1998; Poirier and Wilson, 2006). Of particular interest to this study is that the modeling of both Glass pattern coherence thresholds and RF contor deformation thresholds uses common initial stages of processing. This may, at first, seem surprising since Glass patterns require the accumulation of spatially scattered unconnected local signals while the modulation information in RF patterns is best detected when the patterns form a smooth, uninterrupted contour (Hess et al., 1999; Loffler et al., 2003) and contain repeated regular modulation (Bell and Badcock, 2008; Schmidtmann et al., 2012). Figure 1 reproduces Wilson et al's (1997) schematic of their model and shows the model response to both a Glass (upper) and an RF (lower) pattern. The magnitude of the model response is larger in brighter areas of the plots on the right hand side of the figure and it is clear that the brightest points correspond to the respective centers of rotation^1^. However the common initial stages of the models, which serve to localize the centers of rotation of the two types of pattern, imply that there should be interactions between the two if both are present in a scene and yet this prediction of direct interaction has not been tested.

The current study investigates whether interactions do occur and will present several studies assessing whether the sensitivity to Glass pattern coherence is affected by an overlapping RF contour, whether detection of the location of the center of a Glass pattern is affected by overlapping RF contours and finally whether RF pattern deformation thresholds are impacted by an overlapping Glass pattern. In each case model predictions will be compared to human performance and the conclusion is clear. While the models perform very well in other circumstances they do not predict the pattern of interactions (or lack of interactions) exhibited by human observers and will therefore need revision.

## Method

### General method

#### Participants

Six individuals participated in the experiments (RA, ED, EG, LG, KP, and DM), the latter four of whom were naïve with respect to the purpose of the study. All observers had normal or corrected-to-normal visual acuity. Not all participants observed in every experiment. The project was approved by the University of Western Australia ethics committee and all observers gave informed consent prior to commencing participation.

#### Apparatus

All experiments used code that was written in Matlab 5.3 (Mathworks, Natick, MA, USA) on a Pentium II PC (400 MHz). The computer housed a Cambridge Research Systems (CRS) (Cambridge Research Systems, Kent, UK) 2/4 graphics card which displayed the stimuli on a Hitachi Accuvue 4821 monitor. The screen resolution was 752 × 752 pixels yielding a square field with a side length of 29.5 cm and the screen refresh rate was 100 Hz. Luminance calibration was performed before experimental trials to ensure the specified luminance values were accurate using an Optical OP 200-E photometer (head model number 265) and associated software (Metha et al., 1993). Background luminance was 45 cd/m^2^.

A chin rest was used to maintain a constant viewing distance and at the observing distance of 139 cm the visual angle subtended by one pixel was 1′. The stimuli were displayed in a dark room to minimize reflections and the visibility of other structures and viewing was binocular, except for observer ED who has a squint and was therefore tested monocularly (although both eyes support normal visual acuity). Observers signaled their responses using the left and right switches of a CRS, CB2 button box.

#### Stimuli

The stimuli were Glass patterns, RF patterns, or combinations of both. They were presented on the center of the screen within a 1.7° radius circular region to ensure linear summation of signal over the patterns (Wilson et al., 1997; Dickinson et al., 2009). Although no fixation point was given, observers were informed that the stimuli would always appear toward the center of the screen. The stimulus position was jittered from trial to trial in all experiments, using an additional movement applied to all displayed elements, selected at random from a ± 0.25° horizontal and vertical range, to ensure observers could not reliably use distance to the edge of the screen as a cue. Model simulations did not use this additional jitter.

***Glass patterns (Glass, 1969)***. All Glass patterns (see Figure 1) used in the study contained 36 dot-pairs. The dots had a 2D Gaussian luminance profile (sigma = 2 arcmin; yielding a 4.71′ full width at half height) with a maximum luminance of 90 cd/m^2^ (Weber contrast of one). The dot-pairs were distributed in a pseudo-random manner resulting in a uniform dot-pair density on average (4.13 dot-pairs/deg^2^) and minimizing overlap of dot-pairs. The dots of each pair were separated by 8′ of arc center-to-center. Each dot-pair either represented signal (coherently oriented in a polar coordinate system) or noise (incoherently oriented; pairs oriented at random). Orientation of the signal pairs was defined relative to the center of the Glass pattern. In some conditions the center of rotation of the Glass pattern was moved horizontally (as in Experiment 2), but it was always contained within the displayed pattern and the outer edges of the aperture did not move as a result of this shift.

Three types of Glass patterns were used: (a) concentric, (b) radial, and (c) random. For (a), the orientation of all coherent dipoles was perpendicular to the radial line projecting to them from the center of rotation. For (b), dipoles were oriented along this radial line from the center of expansion. For (c), the dipoles were oriented randomly, representing no global structure.

**Figure 2:**
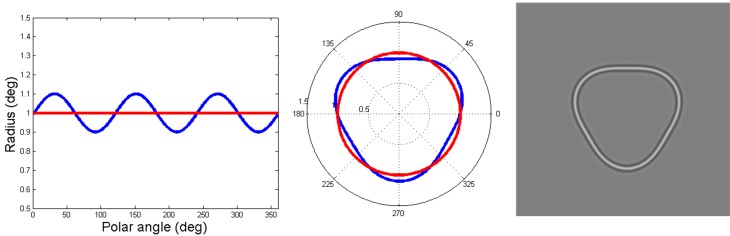
**This image depicts the construction of an RF contour.** On the left, the blue line illustrates the variation in radius as a function of polar angle, showing the amount of deformation of the base circle (the red line). This 0.1 amplitude of modulation to the base circle radius (1°, red contour) creates the RF3 depicted by the blue contour in the middle plot of the figure. The right hand side shows the final pattern when the appropriate luminance profile is applied.

***Circles and RF patterns (Habak et al., 2004; Wilkinson et al., 1998)***. Circles are the particular case of an RF pattern (see Figure 2) where the amplitude of modulation of the radius is zero. The base circle used in the study was a circular contour with a radius of 1°. The luminance profile of a radial cross-section through the pattern was defined by:

(1) $$L\text{​}\left(r\left(\theta \right)\right)=c\text{​}\left(1-4{\left(\frac{r-r\left(\theta \right)}{\sigma }\right)}^{2}+\frac{3}{4}{\left(\frac{r-r\left(\theta \right)}{\sigma }\right)}^{4}\right)e^{-{\left(\frac{r\;-\;r\left(\theta \right)}{\sigma \;}\right)}^{2}}$$

where *r* = radius, *r*(θ)= pattern radius (at θ), *c* = pattern contrast, and σ = 0.0563°. This is not the same as the fourth derivative of a Gaussian (D4) previously used (Wilkinson et al., 1998; Loffler et al., 2003) but this is not critical since global contour integration has also been demonstrated with other profiles with similar σ, e.g., Gaussian profiles (Bell and Badcock, 2008). The revised version does have the benefit of a significant correlation (*r* = 0.91, *p* < 0.001) between its spatial frequency spectrum and that of the Gaussian dot-pairs used for the Glass patterns which should increase the likelihood of cross masking at the early stages of processing and means that lack of masking cannot be readily attributed to spectral differences. The maximum Weber contrast for the RF pattern was 0.5 to ensure that the dots could still be seen when the RF was superimposed on the Glass pattern. All RF patterns used in this study contained three cycles of deformation (RF3). This pattern was selected as Loffler et al. (2003) found that RF3 patterns provide strong global pooling of contour information. To create the RF3 pattern, the base circle was deformed by sinusoidally modulating its radius, *r*_base_, using the equation below such that the radius of the deformed pattern at polar angle θ was:

(2) $$r\left(\theta \right)=\;r_{\text{base}}\left(1+A\;sin\text{​}\left(\omega \theta +\varphi \right)\;\right)$$

where *r*_base_ = unmodulated radius, *A* = radial modulation amplitude, ω = RF (3 in these experiments) and ϕ = angular phase of the pattern.

The degree of deformation of the base circle was expressed as a ratio of the amplitude of modulation to the base circle radius (A in Equation 2), as illustrated in Figure 2. The amplitude was always 0.1 when the RF3 was presented as a mask (see Figure 2). This amplitude was used as it is the maximum value for an RF3 in which there are only three points of maximum curvature (Dickinson et al., 2012). In addition, an RF3 with a modulation amplitude of 0.1 is ~20 times greater than threshold and therefore definitely perceived as an RF and not a circle (Loffler et al., 2003; Bell et al., 2007).

***Combination***. As the dot pairs of the Glass patterns were always at maximum contrast, when they were coincident with the RF pattern, the pixels which would have a contrast greater than one were clipped at one. The impact of this manipulation was incorporated in the model predictions. The Glass patterns' center of rotation and the center of the RF patterns were coincident at the center of the stimulus in all experiments where detection thresholds were determined.

#### Procedure

***Glass pattern detection***. The aim was to measure the coherence threshold for the detection of structure in Glass patterns. A temporal two-interval forced choice (2IFC) paradigm was utilized in which the observers' task was to indicate which interval contained the Glass pattern with structure (the reference was a Glass pattern composed of randomly-oriented dot-pairs). Observers pressed the left switch of the button box if the signal was present in the first interval and the right if it was present in the second. The order of presentation of the stimuli was randomized within a trial, and each pattern was presented for 160 ms with an inter-stimulus-interval (ISI) of 500 ms. Auditory feedback (a tone) was given to ensure optimum performance. Practice trials were run to familiarize observers with the stimuli and response procedure and concluded when the observer indicated they were comfortable with the task.

The method of constant stimuli (MOCS) was used to control presentations. On each trial the signal percentage of the test pattern was set at one of nine levels that were randomly interleaved from trial to trial. Each experimental run comprised 180 trials. Conditions were interleaved, counterbalanced across runs and rerun three times over several days in short testing sessions producing a total of 540 responses per condition (i.e., 60 trials per point). A cumulative Gaussian was fit to the data using non-linear regression (Prism 4.0, Graphpad Software Inc., 2005). The fitted equation was:

(3) $$Y=0.5+0.25\left\{1+erf\left[\frac{x-△}{\sigma \sqrt{2}}\right]\right\}$$

where *Y* = proportion correct, erf = error function, *x* = stimulus coherence level, Δ = threshold, which corresponded to the 75% correct performance level, and σ = the standard deviation of the Gaussian.

The stimulus level (*x*) corresponded to the number of dot pairs which were coherently oriented. In all experiments, *R*^2^ for the fitted curves was ≥0.8.

***RF pattern detection***. Deformation thresholds were determined for RF patterns. The thresholds were expressed in terms of *A* in Equation 2. Habak et al. (2004) divided *A* in Equation 2 by the *r*_base_ but since that value is 1° in the current study it is formally equivalent. A temporal 2IFC procedure was used with the observers' task being to indicate which of the intervals contained signal (i.e., radial deformation); the reference was a circle (*A* = 0 in Equation 2). Details of presentation timing, instructions, order of presentation, feedback and practice trials were as specified in subsection *Glass pattern detection*.

Equation 3 was again fit to the data (Prism 4.0, Graphpad Software, Inc., 2005) to determine threshold, where *x* signified the amount of modulation of the RF3 (A in Equation 2).

***Localizing glass patterns***. The observers' task was to localize the center of rotation of a Glass pattern. A single-interval forced choice (SIFC) procedure was employed and the observer was required to detect whether the center of rotation of the Glass pattern was displaced to the left or right of an implied line between two black (Weber contrast = −1) Gaussian dots (4° above and below the center of the test pattern) with a 4.7′ diameter at half height. The outer limits of the Glass pattern aperture remained fixed relative to the reference dots. The stimulus presentation was 160 ms after which the observer responded using a button box. The observer pressed either the left or right switch to indicate the direction that the center of rotation of the Glass pattern was displaced. 500 ms after a response was made the next trial began. Feedback, instructions and practice trials were provided as specified above in subsection Glass pattern detection.

The MOCS procedure was used to control presentations. On each trial, the amount of displacement of the Glass pattern center was set at one of nine linear spatial steps to the left or right (−4, −3, −2, −1, 0, 1, 2, 3, 4 times a scaler) that were randomly interleaved between trials. The size of the scaler varied, according to each observer's ability, to allow for a full psychometric function. Each of the conditions were interleaved and replicated three times in short testing sessions (180 responses per run) producing a total of 540 responses (i.e., 60 trials per point). The data was fit with a cumulative Gaussian (Prism 4.0, Graphpad Software Inc., 2005) using the following variant of Equation 3:

(4) $$Y=0.5\left\{1+erf\left[\frac{x-PSA}{\sigma \sqrt{2}}\right]\right\}$$

where *Y* = proportion of responses to the right, erf = error function, *x* = amount of displacement, PSA = point of subjective alignment, and σ = the standard deviation of the fitted cumulative Gaussian.

The PSA or midpoint was obtained and corresponded to the offset where the stimulus and the reference appeared aligned or, operationally, where the right-hand switch was pressed 50% of the time.

## Glass pattern experiments

Experiments 1 and 3 investigate the extent to which detecting global structure in Glass patterns or deformed contours is altered by the presence of the other pattern type. This behavioral performance clarifies the extent to which texture- and contour-based processing is interdependent and was also compared to that predicted by the outputs of the computational model to determine whether that model provides an adequate account of this performance.

### Experiment 1: detection of coherent structure in glass patterns

**Figure 3:**
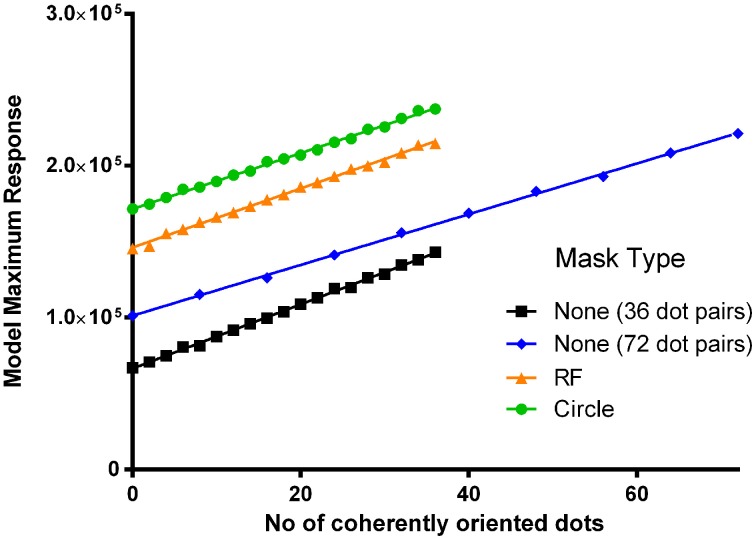
**The maximum response of the model (95% CIs smaller than the symbol) is plotted as a function of the number of coherently oriented dot-pairs in a concentric Glass pattern of either 36 (black) or 72 (blue) dot-pairs.** The response to the 36 pair pattern is also shown when an RF3 (orange) or a circle (green) of 1° base radius (*A* = 0.1 in the RF3) are superimposed.

The first experiment measured the coherence threshold for the detection of structure in a Glass pattern and investigated whether the presence of a circle or RF3 pattern mask alters that threshold. The model (Wilson et al., 1997) was implemented in Matlab 5.3 (using the same parameter set as Wilson et al., 1997) and the dependence of the maximum response on coherence level was obtained when either no mask, an RF3, or a circle was presented coincident with the Glass pattern (see Figure 3, squares, triangles, and circles respectively). Since the model response varies as a function of the specific location of the dot-pairs, the model response data were based on 60 repetitions (independently generated patterns) at each point as the psychophysical data also used this number of trials (95% confidence intervals are smaller than the symbols in Figure 3). In all cases, the model response increased with increasing signal level. Doubling the number of dot-pairs (blue line) produced an elevation in output and a slightly shallower gradient. The addition of an unchanging circle (green line) or RF3 (orange line) pattern mask coincident with the same center of rotation, increased the outputs of the model by a constant amount at all signal levels. Addition of the circle or RF approximately doubled the model response, demonstrating that the Glass patterns and RF patterns were well matched in their model response.

Previous experiments measuring Glass patterns coherence thresholds have shown that when the number of dots is doubled in this low range (from 50 to 100 dot pairs), the threshold number of dots increases with a ratio of ~1.7 (Badcock et al., 2005). The same ratio was obtained from the much more extensive data set of Dickinson et al. (2009). The model also predicts a larger number of coherently placed dot pairs at threshold for the 72 dot-pair stimulus, assuming a constant proportional change in response from that obtained with 0% coherence is required for threshold, since both the response to 0% coherence increases and the slope of the increase in model response is shallower for the 72 dot-pair stimuli. In order to estimate the potential impact of the masks we estimated the proportional change in model response when signal level increases from zero to the threshold level in the 36 dot-pair case. The change in signal level required to produce this proportional change is then determined in each of the conditions.

**Figure 4:**
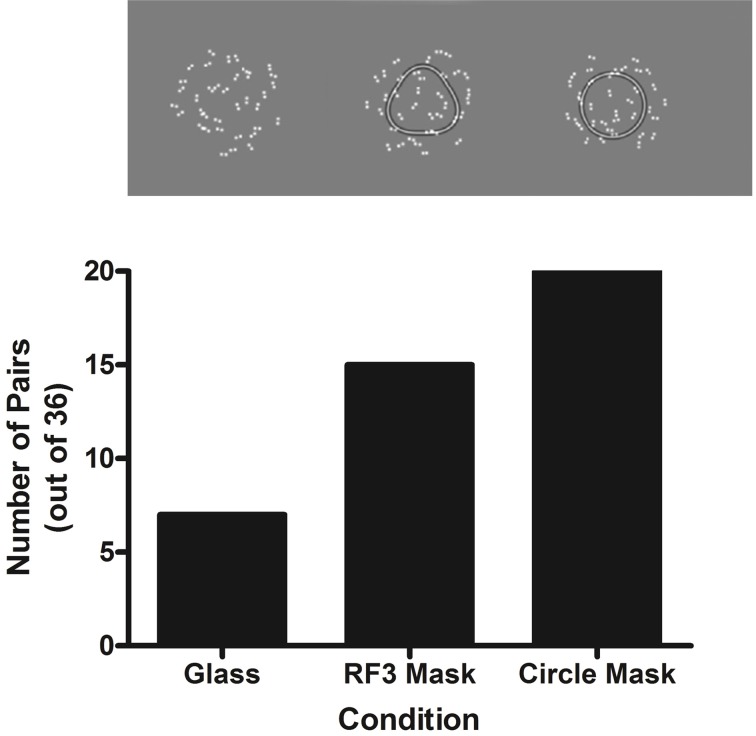
**Upper**: an example Glass pattern with either no mask, an RF3 (*A* = 0.1) or a circle superimposed. **Lower**: the predicted change in thresholds when a mask is added assuming that the no mask condition has a threshold of ~7 (see text for rationale).

Prior work with Glass patterns composed of a small number of pairs yields a threshold of approximately 20% for concentric Glass pattern detection (Wilson et al., 1997; Wilson and Wilkinson, 1998; Badcock et al., 2005; Dickinson et al., 2009). This was used to estimate a threshold of 7 dot-pairs in the 36 pair stimulus. From this estimate, in the no mask condition (Figure 3, black line) the model response when seven coherently oriented dot pairs were presented (maximum response = 81, 165) was divided by the model's response to 0% Glass signal (67,050) giving a proportion of 1.21. Assuming a constant proportional change was required for threshold, this proportion was multiplied by the model response to a 76 dot-pair Glass pattern with 0% coherence and it produced a threshold estimate of 13 dot-pairs which is equivalent to the value of 13.6 derived from the curve fit in Figure 3 of Dickinson et al. (2009), who used a similar procedure to collect the data, and thus supports this method of moving from model response to predicted threshold. The same proportion was also multiplied by the model response to an RF3 mask presented in combination with 0% Glass signal (orange line) and resulted in an increase in the model's response to 176,094; which corresponds to the value obtained with ~15 coherently oriented dot-pairs (i.e., 41.7% coherence level). The prediction for a circular mask yields a greater increase in threshold (from 7 to 20 coherently oriented dot pairs, or 55.6%). This difference in model response between a circle and RF3 mask is expected as the concentric detector model is stimulated less effectively when contours deviate from a circle. The pattern of predicted thresholds is depicted along with the 50% coherence level stimulus examples in Figure 4.

If these relative changes in threshold were detected in observers' results, this would indicate that information from the Glass and RF patterns is amalgamated in a single mechanism as modeled.

#### Method

***Participants***. RA, ED, LG, and KP participated in the detection of concentric Glass pattern cases.

***Stimuli***. Figure 4 displays example test stimuli for the three conditions in the experiment, with each Glass pattern containing 50% signal. The Glass patterns were constructed in the manner described in section Glass patterns. Figure 4 (upper left) shows the condition in which the Glass pattern contained concentric structure with no mask. Figure 4 (upper middle and upper right) depict a Glass pattern with concentric structure in the presence of an RF3 mask or a circle mask, respectively. The masks were constructed as described in section Circles and RF patterns.

***Procedure***. The observers' task was to identify the interval in which the Glass pattern contained signal. The procedural descriptions for this task are given in subsection *Glass Pattern Detection*. The threshold number of dot-pairs was obtained for concentric Glass patterns under three conditions: no mask, a circle mask, or an RF3 (*A* = 0.1) mask.

#### Results

**Figure 5:**
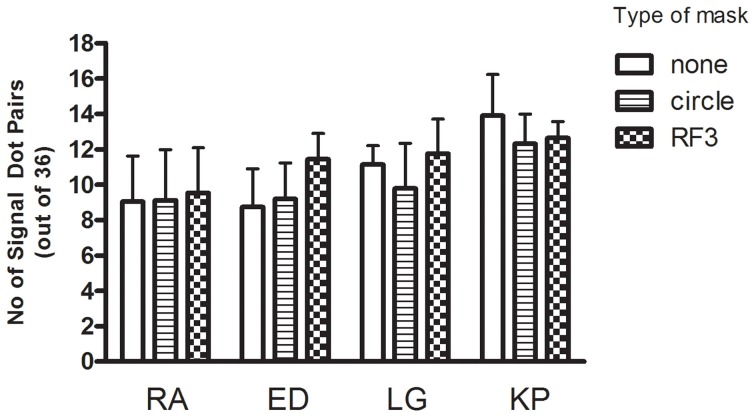
**The threshold (+95% CI) signal required to distinguish a concentric Glass pattern from a random pattern is plotted for each observer in each condition.** There is no significant effect of mask type.

Cumulative Gaussians were fitted to the psychometric functions to obtain 75% correct detection thresholds and these are plotted for each observer in each of the three conditions in Figure 5 with 95% confidence intervals. A repeated measures One-Way ANOVA was performed to detect differences in threshold across conditions. An alpha level of 0.05 was used for all statistical tests in this study.

The differences between conditions are very small relative to the confidence intervals indicating that that the masks had no impact on performance [repeated measures One-Way ANOVA yields no significant effect of condition, *F*_(2, 6)_ = 1.98, *p* = 0.22]. The behavioral data does not correspond to the large masking effects the model predicts.

### Experiment 2: the impact of closed contours on localising the centre of rotation of glass patterns.

**Figure 6:**
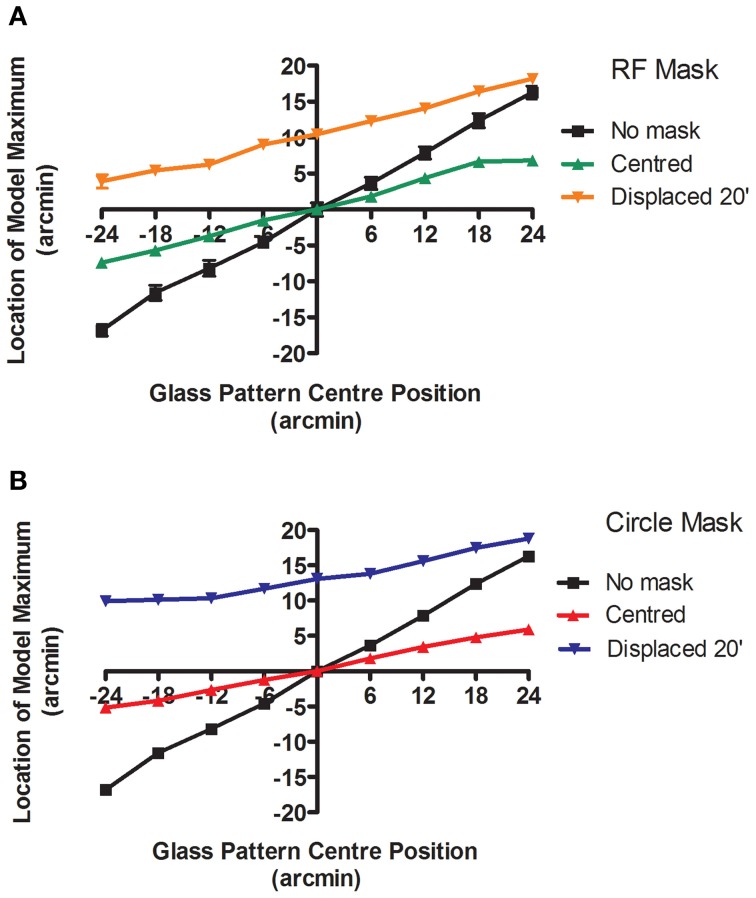
**The location (±95% CIs) of the maximum response of the model is plotted as a function of the center of rotation of the 100% coherent, concentric Glass pattern for three conditions in each plot.** The mask type varies (**A**: RF3 and **B**: circle) and the mask is either centered (green and red) or displaced 20′ (orange and blue). The masks are also detected by the model and provide an anchoring component to the estimated location of maximum response, reducing the rate of change of location as the Glass pattern center position varies and also offsetting the model maximum response towards the center of the mask.

The model predictions in Experiment 1 arise from an output that also identifies the center of rotation of the patterns. Thus in addition to coding the strength of activation it provides a useful cue for localization of the targets. In this experiment the ability of observers to localize the center of rotation is compared to that of the model. The task will be to determine the position of the center of rotation of a Glass pattern relative to two vertically displaced markers. Performance with Glass pattern alone will be compared to that obtained when either an RF3 or a circle is also present. The latter elements will either be centered or displaced to one side by 20′. Figure 6 shows the position of maximum activation in the model's output as a function of the Glass pattern center of rotation. Each point was created by collecting responses to 60 different pattern examples and the mean location is plotted. The difference between the estimated center location for centered and off-centered Glass patterns is assumed to provide the signal the model uses to determine the offset.

The black line shows that the model's output does move with the center of rotation of the Glass pattern, albeit to a smaller extent than the physical displacement. This reduction in apparent shift may arise from the fixed location of the display aperture, relative to the reference markers, which renders the number of pattern elements asymmetric as the center is moved towards the edge of the aperture. This reduced displacement is not critical to the relative predictions across conditions that are of central interest here.

The green and red lines show that adding a centered RF3 or circle, respectively, reduces the magnitude of change in the location of the maximally active point and thus would be expected to produce an increase in the displacement threshold. Finally the orange and blue lines show that adding an RF3 or circle, respectively, displaced 20′ to one side of center, both reduces the gradient and causes an offset in the location of maximum activation towards the location of the mask. Thus if the human visual system employs an equivalent system the additional components should both increase the displacement threshold, relative to the unmasked case as a larger offset is required to produce the same change in model output, and also produce a displacement of the point of subjective alignment. While precise quantitative predictions could be employed qualitative predictions will suffice to evaluate the model's performance in this case. Observers were tested using exactly the same stimuli as those employed to produce the predictions.

#### Method

***Participants***. RA, ED, EG, and DM observed in this experiment.

**Figure 7:**
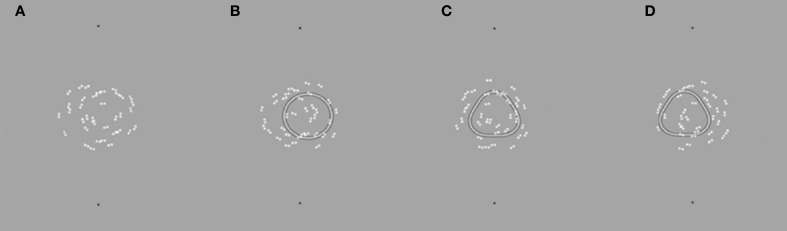
**Example stimuli are depicted as described in the text.** Judgments were made regarding the center of rotation of the Glass pattern relative to the black outer Gaussian markers.

***Stimuli***. All Glass patterns presented in this experiment were concentric and contained 100% signal. Example stimuli are presented in Figure 7. Figure 7A illustrates a Glass pattern with no mask. Figure 7B shows the condition with a circle mask displaced 20′ to the right. Note that the model suggested that the center of rotation of the Glass pattern would be perceived to be displaced in the same direction as the circular mask. Figure 7C shows the condition with a centered RF3 mask and the center of rotation of the Glass pattern to the left of the two reference dots. Stimulus details are described in full above as is the procedure. Figure 7D shows a Glass pattern with the center of rotation to the right and an RF3 mask displaced 20′ to the left.

Observers were required to localize the center of rotation of a Glass pattern under eight conditions: no mask (this condition was run twice for counterbalancing purposes; an RF3/circle centered in alignment with the black reference markers; displaced 20′ to the right; displaced 20′ to the left.

#### Results

**Figure 8:**
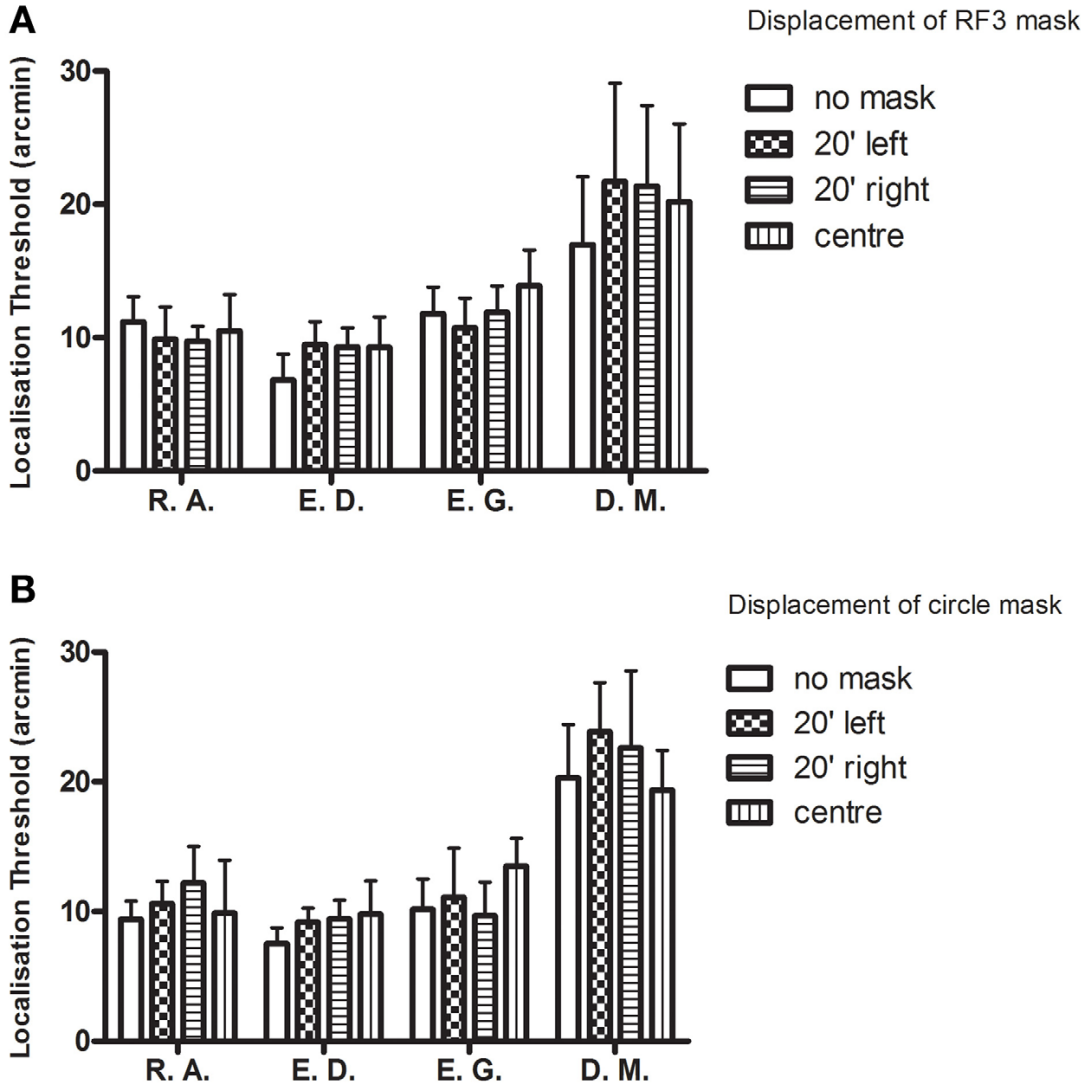
**Localization thresholds (+95% CIs) are plotted for each observer in each condition.** The addition of a mask (**A**: RF3 and **B**: circle) had no consistent impact on localization precision.

To address the first prediction, sigma values were examined, that is, the standard deviation of the Gaussian fit to the distribution (see Equation 4). These values were graphed for each observer for the RF3 and circle mask conditions (see Figures 8A,B, respectively).

The model predicted larger values when a mask was present than when it was not. The differences between conditions for each observer are small and most confidence intervals for each observer overlap. Two repeated measures one-way ANOVAs were performed to detect whether sigma levels were significantly different across the various masking conditions. The values did not vary for either the RF3 [*F*_(3, 9)_ = 1.15, *p* = 0.38] or the circular mask conditions [*F*_(3, 9)_ = 1.17, *p* = 0.38].

**Figure 9:**
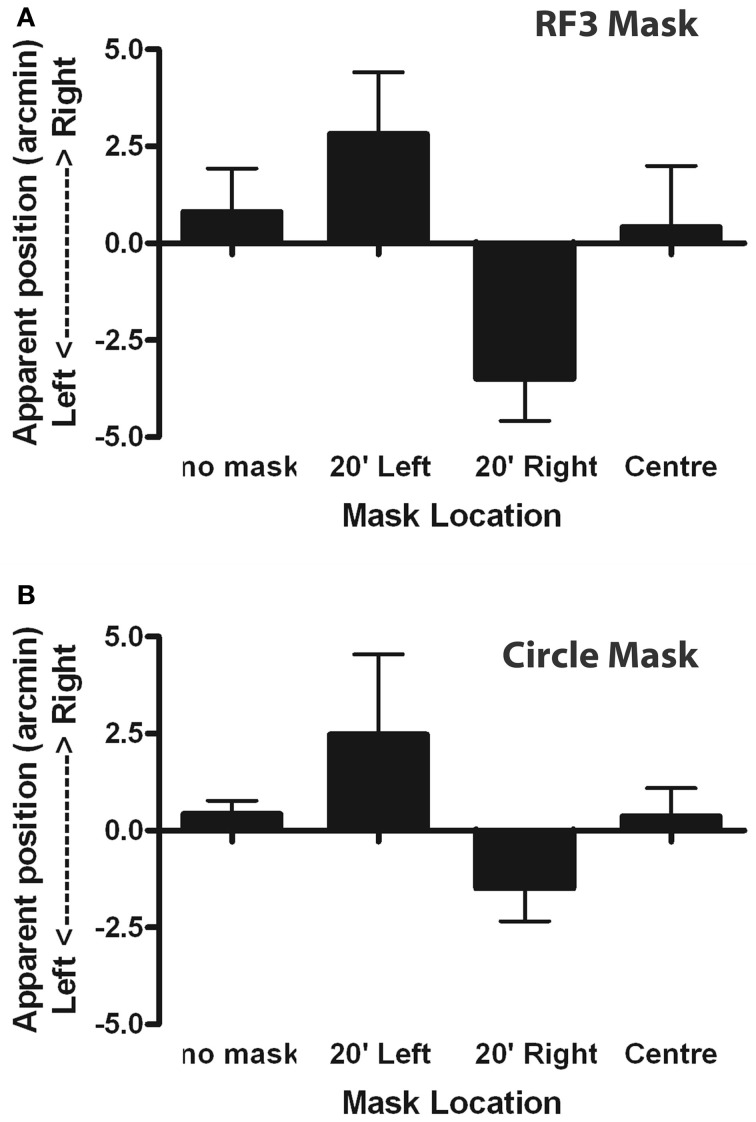
**The average perceived shift in Glass pattern center location is plotted (±95% CIs) for the group as a function of masking condition.** The means are offset in the opposite direction to the displaced masks although this effect is only significant in the RF3 **(A)**, and not the circle **(B)**, conditions.

To test the second model prediction, the PSA was examined. The PSA (derived from the best fit of Equation 4) signified the position where the stimuli appeared aligned with the reference. Summary group data are presented in Figure 9 which plots the perceived shift of PSA from veridical as a function of condition, averaged across observers. All observers gave the same pattern of variation across conditions, although one observer had a response bias to one side of centered in all conditions. In the RF3 mask conditions, the repeated measures one-way ANOVA revealed that the PSA values were significantly different [*F*_(3, 9)_ = 23.09, *p* < 0.05). A Newman–Keuls Multiple Comparison Test detected (*p* < 0.05) that PSA of both the 20′ left displacement condition and the 20′ right displacement condition were significantly different from the PSA of the remaining conditions. No significant differences were observed between the PSA of the no mask and the RF positioned in the center conditions. Although the same trends in PSA values observed in the RF3 mask conditions were observed in the circle mask conditions, the differences in PSA values for the circle mask conditions were not statistically significant [*F*_(3, 9)_ = 2.36, *p* = 0.14].

#### Discussion

This experiment examined position coding, and specifically, tested whether the impact, on localising the center of rotation of a concentric Glass pattern, of a set of mask patterns, matched that predicted by the model. The results do not match. The model predicted firstly that localization thresholds would be increased when a mask was present. However, the presence of an RF3 or circle mask did not influence the displacement thresholds, indicating that observers were equally competent in accomplishing the task with a mask present. This lack of masking suggests that the processing of concentric Glass patterns and low RF patterns employ independent mechanisms. The model also predicted that the perceived position of the composite structure would be displaced in the direction of the circle or RF3 pattern mask center. The findings from this experiment reveal the opposite effect with an RF3 mask (and a non-significant effect in the same direction for circle masks). A control experiment also established that the removal of trial-by-trial feedback made no significant difference to this result. The PSA values for each of the displaced RF3 masking conditions were significantly different from the PSA values for the remaining conditions, with the displacement in the opposite direction to that predicted by the model.

### Experiment 3: detection of deformation in radial frequency patterns

The third experiment is the complement of Experiment 1 in testing whether the presence of a Glass pattern mask alters the detection of deformation in an RF pattern. In this case it is necessary to generate model predictions differently. This set of predictions is based on the version of the model tailored to detect deformation in RF patterns (Poirier and Wilson, 2006) which provides an RF number specific level of activation by detecting periodic variation in curvature and then cross-correlating that variation with sinusoidal functions at different frequencies. In this case the predictions were initially based on the level of activation change the masks induced at RF3, since the target was an RF3 pattern.

**Figure 10:**
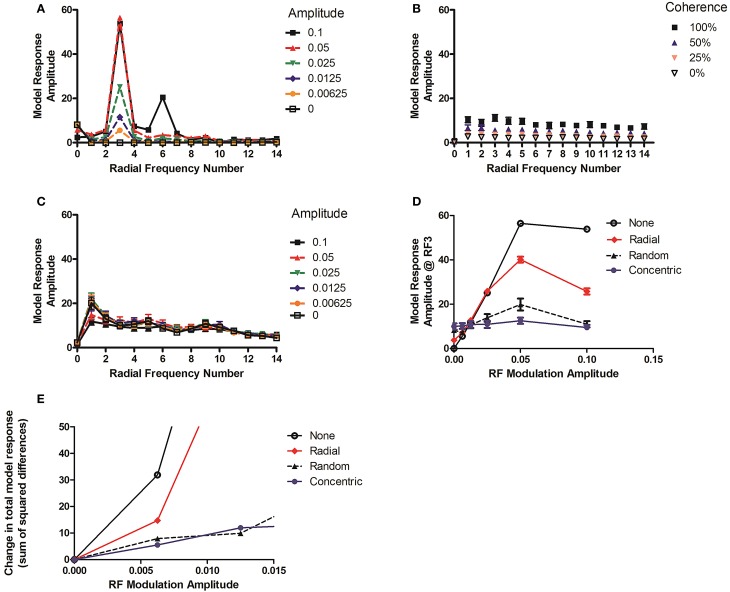
**(A–C)** depict the models response at each radial frequency as a function of signal level. The plots show results for **(A)** RF3 alone, **(B)** concentric Glass pattern alone, and **(C)** an RF3 with a 100% coherent 36 dot-pair concentric Glass pattern superimposed. **(D)** Shows the change at RF# 3 in **(C)** when the amplitude is varied with a concentric Glass pattern overlapping (blue) and when a random Glass pattern (black), a 100% coherent radial Glass pattern (red) or no Glass pattern superimposed (as in subplot a). **(E)** Shows the change in overall response as amplitude varies, quantified as the sum of the squared differences at every RF#, for the same mask conditions.

In order to keep the procedure as similar as possible in the conditions containing Glass patterns the average peak model responses for 60 different examples of the Glass stimuli are calculated. However, the response for the RF3 pattern was only generated once since there is no variability in the response to the contour from one occasion to the next, even though the phase was randomized. Figure 10A shows the model response at each RF as the amplitude of the RF3 pattern was varied.

The model response amplitude at RF number (RF#) 3 increases with deformation level, in the range depicted. The output of the model to concentric Glass patterns grows as the coherence level increases but does not vary substantially across RF# (Figure 10B). Adding a 100% coherent Glass pattern to the image causes a substantial reduction in both the model's response to the RF3 and in the variation at RF3 when amplitude level increases (Figure 10C). Figure 10D depicts the amplitude at RF# 3 as the modulation level of an RF3 pattern is varied in the range that covers the thresholds obtained behaviorally in Loffler et al. (2003) and Bell et al. (2007).

Thresholds would be expected to be proportional to the amount of change in model output over this range if the model is to explain performance. The response as a function of modulation amplitude is shown for four different simultaneous mask conditions. The largest reduction in response variation occurs when adding a concentric Glass pattern (100% coherent; filled circles). This pattern should therefore produce most masking. A random Glass pattern (0% coherent; filled triangles) also has a substantial, albeit slightly reduced, impact and thus should also produce significant masking. A radial Glass pattern (100% coherent; red diamonds) was also used. As this is not a circular structure it has little impact on the response until quite large RF amplitudes and should produce less masking.

An alternative approach is to allow the differences to be detected at any RF number, rather than just at RF3 since the impact of the mask is to change responses at many frequencies. To estimate this, the sum of the squared differences between the stimuli with amplitude modulation of 0 and those with additional modulation was calculated (see Figure 10E). The predictions are similar. Both the random Glass mask (Random, black dashed lines, triangles) and the 100% concentric Glass (Concentric, blue line, filled circles) suppress the change in response as modulation amplitude increases relative to the unmasked RF3 (None, black line, open circles), with the radial Glass (Radial) predicted to produce a weak and intermediate effect.

#### Method

***Participants***. RA, ED, KP, and LG observed in this experiment.

**Figure 11:**
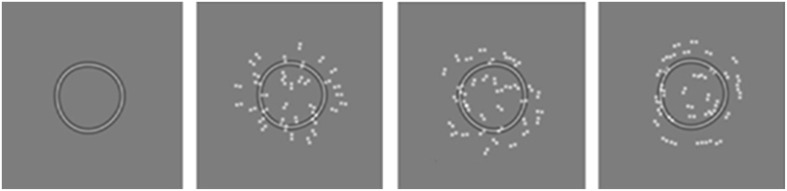
**Example stimuli in which RF deformation must be detected as a function of masking conditions employing no mask, a radial, random, or concentric Glass pattern mask (left to right respectively)**.

***Stimuli***. The critical details of the stimuli are provided in the general method section above. In this experiment an RF3 is the target and it varies in modulation amplitude (A in Equation 2). There are four conditions (depicted in Figure 11 with *A* = 0.018); (1) the RF3 without a mask, (2) the RF3 with a 100% coherent Radial Glass pattern mask, (3) the RF3 with a random (0% coherent) Glass pattern and (4) the RF3 with a 100% coherent Concentric Glass pattern mask. Targets and masks were always centered on the same position in this experiment.

***Procedure***. A 2IFC paradigm was employed in conjunction with the MOCS. The observer's task was to identify in which of the two intervals the RF pattern had the most amplitude modulation with one interval always containing a circle (*A* = 0). The details are given in section RF pattern detection.

#### Results

**Figure 12:**
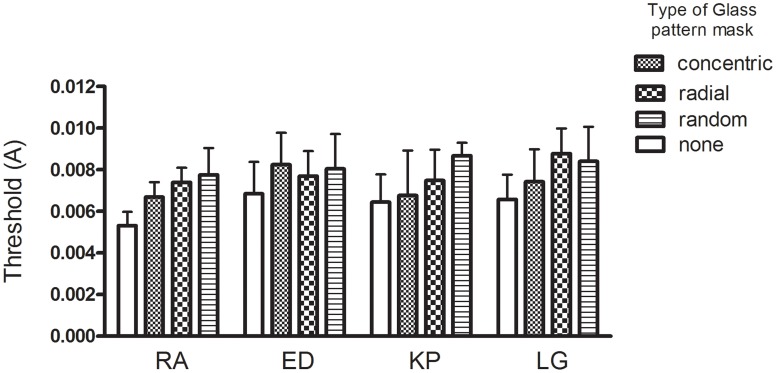
**The amplitude required for deformation threshold (+95% CIs) is plotted for each observer with either no mask or a superimposed Glass pattern with radial, concentric, or random structure.** Overall the masks were equally effective in reducing performance.

The threshold amplitudes (+95% CIs) are plotted for each observer in each condition in Figure 12. There is a trend for the thresholds for the no mask condition (Figure 12, vertical lines) to be consistently lower than the other three Glass pattern masking conditions (Figure 12, diagonal lines, checks, and horizontal lines). However the confidence intervals overlapped in several of the masking conditions and thus a repeated measures One-Way ANOVA was performed. This revealed a significant main effect of condition type [*F*_(3, 9)_ = 11.80, *p* < 0.05]. To detect in which conditions significant differences were found, a Newman–Keuls Multiple Comparison Test was conducted and revealed that the no mask conditions had significantly lower thresholds than the other conditions (*p* < 0.05). However, the different masking conditions were not significantly different (*p* > 0.05). That is, the concentric Glass patterns were no more effective as a mask than either radial Glass patterns or randomly oriented dipoles.

#### Discussion

As noted earlier, the computational models predict quite different masking strengths with the different masks employed. The pattern of results obtained is inconsistent with these predictions showing equivalent masking in all conditions. This is most readily explained at the local level of analysis since dot-pairs crossing the RF contour occur in all conditions and these may have obscured the amplitude modulation to some extent. The task was still possible however and if an interaction had occurred at the global level of analysis substantial effects would have been expected. The results do not support common processing of RF contours and Glass patterns at the global level of analysis.

## Discussion

The aim of this sequence of experiments was to determine whether global form defined by texture orientation interacts with global form defined by a continuous contour when processed by the human visual system. Previous work has made it clear that shapes can be delineated with both cues (e.g. Biederman and Ju, 1988; Pasupathy and Connor, 2002; Habak et al., 2004; Wilson and Wilkinson, 2004; Loffler, 2008; Dickinson et al., 2009), although edge cues are more important for defining shape than surface properties (Biederman and Ju, 1988; Elder and Zucker, 1998). However, a system that can exploit both cues might do so independently or, instead, combine both signals to produce a common solution. In the latter case interactions might be anticipated.

A second reason for conducting this series of experiments was to test the limits of an otherwise very successful model of the processes encoding the textures signaled by Glass pattern coherence (Wilson et al., 1997; Wilson and Wilkinson, 1998) and separately the deformation of circular contours represented by RF patterns (Rainville and Wilson, 2005; Poirier and Wilson, 2006). The initial stages of the models proposed for these two patterns are the same and they provide responses which clearly indicate the center of rotation of both pattern types and which are proportional to the coherence level in Glass patterns and the modulation amplitude in RF patterns. Thus interactions between the two pattern types were predicted by all of the model variants, although the performance of the models in this context has not previously been considered. There is prior data examining how RF contours interact in a lateral masking context (Habak et al., 2004; Bell and Badcock, 2008) which shows strong masking when the contours are in phase and Habak et al. have considered how the model could accommodate that result but the cross pattern interactions have not been examined.

The outcomes of the sets of experiments are clear. The behavioral data do not correspond to the predictions generated from the models. The contour patterns did not impair the ability to detect coherent structure in the Glass patterns (Experiment 1) and while adding the Glass patterns did impair detection of deformation of the contours in Experiment 3, the impairment was consistent with local pattern interactions between dot-pairs and the contour and not consistent with the mask pattern specificity predicted by the model.

This interaction at the local level of analysis would be expected to occur when RF contours mask Glass patterns as well. However a single RF contour only covers a small fraction of a Glass pattern and this probably renders the effect too small to observe with the current stimuli. Interactions would be expected to be stronger with multiple rings of RF contours that covered a whole Glass pattern. However, these interactions between local elements could induce local tilt illusions (Dickinson et al., 2010, 2012) which would be likely to flow on to distort the global structure and are therefore not of central interest here because this would be a distortion at the local rather than an interaction at the global level of processing. The proposed models of global processing being evaluated predict substantial masking effects with the stimuli we have employed and the behavioral data does not show those effects. Finally, the localization experiment showed no effect on the thresholds for localizing the center of rotation of the Glass pattern when contour stimuli were added, even though the model predicted a significant reduction in sensitivity. The localization performance was comparable to previous estimates for unmasked Glass patterns (Harvey and Braddick, 2008; Dickinson and Badcock, 2009) and thus the lack of mask effect on precision cannot be attributed to poor overall performance levels.

The model also predicted a significant shift in the perceived location of the center of rotation towards the center of the added contour (Figure 6) but Experiment 2 obtained a significant shift in the opposite direction in all cases. This outcome is reminiscent of previous research examining the interaction between features in localization experiments which have shown strong repulsion effects between separately defined objects (Kohler and Wallach, 1944; Ganz and Day, 1965; Ganz, 1966; Badcock and Westheimer, 1985a,b) implying that the Glass patterns and the RF contours are extracted as separate entities with their own assigned locations and that repulsion occurs between these perceived locations.

Thus the results suggest that global textural coherence and global contours are extracted by separate processes within the visual system. This is, perhaps, not a surprising conclusion given previous work showing different abilities to combine information defined by increments and decrements with the two patterns (Wilson et al., 2004; Badcock et al., 2005; Bell and Badcock, 2008) but it does require a substantial revision of the extant models proposed to account for performance with those stimuli. As has been shown, the models do predict interactions which do not occur. What is needed is a method for restricting the inputs to one pattern type or the other, so that separate processing can occur. Restricting the operation of the computations to annuli, and then selecting the most informative annuli rather than whole stimuli could allow separate operation but the processes involved in finding the center of the two pattern types (and particularly low-coherence level Glass patterns) must be distributed. This should lead to the errors in the estimation of center location predicted above and those errors did not occur behaviorally. While this does still seem like a fruitful direction to explore it is not readily apparent how the current models can be altered to allow this, while retaining the other characteristics that have already accounted for a considerable array of data (Wilson and Wilkinson, 2004; Loffler, 2008).

### Conflict of interest statement

The authors declare that the research was conducted in the absence of any commercial or financial relationships that could be construed as a potential conflict of interest.

## Footnotes

^1^We thank Hugh Wilson for providing the Matlab code implementing the Wilson et al. (1997) and Poirier and Wilson (2006) models for use in this research.
